# Beyond early warning: towards greater granularity in the use of event-based surveillance for public health emergencies

**DOI:** 10.1186/s12889-024-20963-2

**Published:** 2024-12-18

**Authors:** C. J. McKnight, A. T. Aboushady, C. R. Lane

**Affiliations:** 1 WHO Health Emergencies Programme, WHO Regional Office for Europe, Copenhagen, Denmark; 2 WHO Health Emergencies Programme, WHO Country Office in Yemen, Sana’a, Yemen

**Keywords:** Emerging infectious diseases, Neglected tropical disease, Infectious disease outbreaks, Outbreak response, Time to detection, Time to notification, Medical countermeasures, Event-based surveillance, Early warning alert and response system, WHO disease outbreak news, ProMED mail

## Abstract

**Background:**

The international health emergency caused by the emergence of the SARS-CoV-2 virus demonstrated the expanding usefulness of multi-country disease outbreak information gathered through event-based surveillance (EBS) as an extension beyond the main purposes of early warning, alert, and response (EWAR). In this article, previous events of multi-country outbreaks from 2010–2019 were reviewed for how EBS, within an expanded sphere of Epidemic Intelligence (EI), may help to enhance the understanding of outbreaks for a more timely and nuanced, multiple-point trigger approach to health emergencies.

**Methods:**

The public, open-source database of ProMed reports were reviewed for the date of first notification on major outbreaks of infectious diseases and then compared for subsequent dates of any new, exceptional epidemiological findings (novel host, settings, transmission characteristics) as a determining factor for prolonged, multi-country events later acknowledged on the WHO disease outbreak news (DON) website, or by peer-reviewed journal publication if no related DON information became available.

**Results:**

During the preceding decade, there was an ongoing occurrence of unexpected outbreaks requiring new information about previously unknown pathogens, such as MERS-CoV, and longstanding threats from multiple neglected tropical diseases. During these international outbreaks, key scientific insights about new host species, viral persistence, occurrence of human-to-human spread, and transmission setting, became known over the course of the response.

**Conclusion:**

The timeliness between initial alerts of early outbreak detection and key epidemiological evidence about the emerging threat reached far beyond the first warning for the global community. To improve on the best knowledge available for an immediate response, it is recommended that further gathering and documentation from event-based surveillance is engaged to create a more complete assessment for uncontrollable infectious disease outbreaks and epidemics. Enhanced EBS (through modern tools, e.g., Epidemic Intelligence from Open Sources (EIOS) are critical for timely detection and response to such events.

## Introduction

At the onset of the COVID-19 pandemic, while many unaffected countries were duly informed with urgent warnings about the global threat from the novel virus in humans, the public health responses were not sufficiently and rapidly adapted for the impending national emergency [1, 2]. It has been noted that not every country heeded the first warnings with a robust response. While the initial alerting messages about new disease detection were essential, recognition of other key trigger events is important for improving the response and theoretically ending outbreaks sooner with this knowledge. This experience suggests that adequate information specific to the disease threat, geographic relevancy, and in the given context, could have benefitted the decision and intervening actions taken by countries [3].

It has been said that “The first few days are often the most important to prevent outbreaks from becoming epidemics and pandemics; by warning the world early, countries can access resource stockpiles, funding mechanisms, and decision support tools; coordinate epidemic response and surveillance across borders; and prepare the public for imminent danger” [4]. The assumption is that early detection of outbreaks will stop transmission early enough, with the information needs adapted as necessary for the response. However, exceptionally challenging outbreaks which remain active and spread to a multi-country level of significance should also be assessed for additional aspects of consideration by use of event-based surveillance. One of the greatest challenges to responding may be the lack of knowledge about the emerging disease threat. And, as was experienced over the COVID-19 pandemic, the rapidly evolving situation required continual situational analysis and risk assessment at all levels of government to mount the most appropriate response [2].

WHO held a technical workshop on event-based surveillance (EBS) in 2013 and later summarized the methodology and uses in a guidance document for countries to strengthen public health emergency operations centers. This document focused on early warning, alert, and response (EWAR) uses, and the methods for Epidemic Intelligence (EI) practices [5]. With technological enhancement since then, a significant number of countries have implemented EBS at the community and health facility levels primarily with an EWAR focus to address prioritized threats. As well, considering the increasing digitalization of disease information around the globe and the growing need for real-time updates, the core aims of EBS for EWAR shifted and were succeeded by routine and continual updates for EI to make adjustments in response activities. EBS expanded to encompass more than the routine monitoring of indicators and automated thresholds for action but rather on the screening of all available information to detect any event happening in the community.

The importance of robust information sharing and reporting on disease events is emphasized in the International Health Regulations (IHR 2005) within the framework towards international disease control and reporting on potential Public Health Emergencies of International Concern (PHEICs) [6]. In instances where countries have delayed notifications or provided limited details, additional information sources are necessary for effective response efforts. IHR allows the WHO to consider reports from unofficial sources, including scientists, social media, non-governmental organizations (NGOs), newsletters, and broadcast outlets [7]. Through the collection of EI and ongoing analysis of outbreaks, WHO seeks to obtain verification from the affected countries and shares with Member States for a robust global response [8].

In prior research, the benchmark of event-based surveillance systems has been to determine the quantifiable gains in achieving a rapid public health response, through the shortening of the number of days from outbreak onset to formal notification with the verification on reported events [9, 10]. This indicator can be readily determined from numerous sources of reports and comparable across outbreaks. After the first official notification for a new disease incident, the usefulness of EBS becomes less obvious and common in utility. This review of outbreak experiences addresses the EBS uses over the evolution of prolonged outbreaks to better construct an understanding of how information updates show benefits over time. This study evaluates historical outbreaks for clear and distinct turning points when event-based surveillance was important and documented in granules on reporting systems after the point of early warning and alerting.

## Methods

### Study type

This study is a retrospective review of database entries in the Program for Monitoring Emerging Disease (ProMED) mail system from January 2010 through December 2019 for reports about significant emerging, infectious disease outbreaks which occurred in multiple countries.

ProMED is an online platform launched by the International Society for Infectious Diseases (ISID) in 1994. ProMED monitors and shares information on global health and disease outbreaks. ProMED operates through a network of volunteers who collect and disseminate news, updates, and analysis from various sources. Its main objective is to provide early warnings, identify disease outbreaks, and share valuable information to support rapid response and control measures. ProMED has a diverse subscriber base, including health professionals, researchers, policymakers, and journalists worldwide. It has been crucial in monitoring and reporting significant outbreaks, contributing to global awareness and collaboration. It has been at the forefront of reporting significant and minor disease outbreaks and biothreats. They were the first public network to provide information on notable outbreaks requiring international responses such as SARS, new spillovers of filoviruses, and numerous other recent microbial threats. For this retrospective study of open-source outbreak data, it was not appropriate or possible to involve patients or the public in the design, or conduct, or reporting, or dissemination plans.

### Search approach

Manual review of the ProMED database was conducted based on the following inclusion criteria; (1) reports about the first occurrence of new, unusual and/or unverified outbreaks affecting human health, (2) outbreaks of multi-country importance, and (3) if new, key event triggers were identified which were significant epidemiological findings concerning the dynamics of the outbreak. Data extraction was completed by saving the text and web addresses for the included reports in Microsoft Excel and Word files.

These follow-up events were then measured for timeliness of formal reporting on WHO Disease Outbreak News (DON), or peer-reviewed journal publications if no DON entry was found. DON is a WHO platform that provides timely and reliable information about confirmed acute public health events and/or potential events of concern. It is an official source of updates on emerging or re-emerging infectious diseases and public health events worldwide. DON aims to disseminate verified information obtained from official sources, enabling governments, healthcare professionals, and the public to stay informed and take necessary actions to prevent and control the spread of diseases. The timeliness of reporting was measured by calculating the time difference between the ProMED entry and the recognized reporting source during the response phase.

### Exclusion

For follow-up information on outbreaks, grey journal publications and social media posts did not fulfill the above criteria as formal or recognized sources of outbreak information and were excluded. Furthermore, although molecular surveillance databases like GISAID have become more prevalent and valuable sources of information following the SARS-CoV-2 pandemic, their data is not incorporated into this analysis. These databases primarily focus as unique surveillance platforms that track genetic variants of existing pathogens and provide technical laboratory findings on new changes in antigenic properties. It is important to note that occasional findings from these databases may be published or reported through media channels accessible via event-based surveillance. However, this review has not included such information for the specified purpose.

## Results

During the period from January 1, 2010 until December 31, 2019, 29,933 new and updated posts were retrieved from the main ProMED-mail web database. A considerable portion of the analyzed posts consisted of follow-up entries of ongoing outbreaks in specific countries or regions. These posts typically took the form of continuous entries, providing periodic updates with the latest available data on reported cases and fatalities. Additionally, moderators supplemented the information with valuable insights, historical perspectives, and contextual details to enhance the understanding of the outbreak situation.

For disease outbreaks which resulted in cases in multiple countries, after the initial notices about the new event, the manual review of entries found additional updates concerning important epidemiological findings across the stages of outbreaks. In this review, a total of 7 early alerting events were identified which had additional granules of information captured later with the event-based surveillance approach. The events are classified by type of trigger, described in Table 1. After the initial alerting function providing an early warning, an event-based surveillance method, via ProMed, reported important events about determining factors; which often supplemented epidemiologic investigations with definitive laboratory investigation, revealed unknown animal spillover linked to human exposure, a new transmission pattern, setting or region, identification of unreported sequalae events, counter-measure effectiveness, and recurrent warning after the outbreak was assumed to be finished.

**Table 1:** Summary of multi-country outbreak events with additional EBS granules after early warning

Pathogen	Year	Directly-affected countries	Exceptional event trigger type	Description of event	Time between first detection and formal notification about trigger event (days)	Report references
Dracunculiasis	2011	Chad, Angola, Cameroon	Novel host linked to transmission to humans	Guinea Worm naturally occurring non-human infections in dog reservoir	876	[11–15]
MERS-CoV	2012	Jordan, Saudi Arabia, the Republic of Korea	Context-specific characteristics	Air contamination and long-range transmission amplification in nosocomial infections; Discovery of viral persistence in immunocompromised patient	1411 (airborne transmission); 1706 (viral persistence)	[16–22]
Ebola virus	2014	Guinea, Sierra Leone	Counter-measure effectiveness	Safety and effectiveness of ring vaccination in stopping new flare-up, due to viral persistence	1369	[23–28]
Nipah virus	2014	Philippines, India	New geographical spread of Henipavirus	Expanded to regions with first human-to-human transmission, following prior annual occurrences in Bangladesh	1450	[29–33]
Zika virus	2015	Brazil, French Polynesia	Late disease sequelae after inconclusive outbreak identification	Presumed Dengue / Measles outbreaks, resulting in GBS and Microcephaly	205 (Microcephaly detection); 285 (GBS)	[34–42]
Lassa Fever	2016	Togo, Germany	New setting for outbreak	Secondary transmission in modernized healthcare setting from a newly-discovered strain	9	[43–45]
Mpox	2019	Nigeria, Singapore	Recurrent warning	Imported sentinel case after outbreak period ended	586	[46–48]

In chronological order of disease outbreaks reports, beginning in 2010, an unexpected outbreak of Guinea Worm occurred in Chad, which had been previously certified for disease eradication. Within the following years, further investigation revealed that for the first documented time, infected dogs were the reservoir species of *D. medinensis* worms and a cause of human outbreaks. In 2018, Angola detected cases with similar disease etiology, as well as Cameroon with a human case and infected dogs near the border of Chad. The gap between the first report recognizing the unusual cases of Guinea Worm and the follow-up evidence about the new transmission pathway elapsed over several years of valuable response in the global eradication efforts. By annual totals, there were approximately 57 human cases reported in the time between initial alert and formal notice by publication.

Following this unique event finding, in 2012, early reports about a cluster of atypical pneumonia cases in an ICU hospital in Zarqa, Jordan, were identified as caused by MERS-CoV, that was newly discovered in a case in Saudi Arabia later that year. After the initial detection, many hospital outbreaks were reported in Saudi Arabia and surrounding countries. Only after further epidemiological investigations in 2015 in the Republic of Korea, during a large outbreak linked to an imported case, in-depth clinical studies found two unreported drivers for disease transmission of the Beta-Coronavirus: Persistence of the virus can occur in patients, and also the virus spread through long-range air-borne transmission in medical care settings as well as close contact [49, 50].

The clinical significance of viral persistence was also a new finding for Ebola Virus Disease following rare cases discovered in the aftermath of the West Africa epidemic from 2013–2016. During the response, the development of an effective medical counter-measure with the new rVSV-ZEBOV vaccine, was field tested in Guinea and Sierra Leone, showed ring vaccination as a new defense in containing these unforeseen flare-ups of the virus. The importance of this new proven response strategy reported in 2016 was crucial afterward for effective ring vaccination efforts in Democratic Republic of Congo (DRC) in 2018–2019 to control challenging new outbreaks in affected communities.

The emerging threat of Nipah virus was well documented from prior outbreaks in a few South-East Asian countries, surrounding the Bay of Bengal, such as Malaysia and Bangladesh, which observes annual outbreaks linked to the consumption of palm date sap frequented by fruit bats. In 2014, the risk zone for Nipah widened to include The Philippines. In this outbreak, human-to-human transmission chains were documented for the first time outside of Bangladesh. Although human cases of Henapivirus was documented in the preceding decade in Malaysia and Australia (Hendra virus), control efforts were less involved because it was not known to spread greater in human populations than the sporadic spillover from animal hosts. However, within 4 years of The Philippines cases, another incident of human-to-human cases was identified in a new outbreak foci in Kerala, South India. Fruit bats with the virus were also found in Thailand in 2010, although this finding was not known until after The Philippines outbreak and there were no human cases detected there.

Zika virus spread in Brazil during 2014–2015 after the first reports noted presumed, yet unconfirmed measles and/or Dengue cases in affected populations. These incidents were then followed by the detection of the new virus in the continent in April 2015. More than half a year elapsed since the initial reports of the disease outbreaks when an alarming increase was found among infants born with congenital birth defects characterized by microcephaly. A similar association between Zika infection in pregnant mothers and Congenital Syndrome in newborns was later made in French Polynesia retrospectively for an epidemic that occurred in 2014. Another temporal link between Zika infection and Guillain-Barré syndrome (GBS) cases was made in February 2016, significantly raising the importance of disease control and prevention efforts in impacted countries with epidemics. Human-to-human transmission through sexual contact was also identified; although occasionally occurred, it was not to be a factor sparking nor driving outbreaks.

When countries delay recognition of novel disease outbreaks, the consequences are recognized later elsewhere. For the undetected circulation of Lassa Fever in Togo, the first sign was reported in an imported, returning traveler case in the United States. This case was rapidly reported in March 2016, and nine days later another country, Germany, experienced for the first time an incident of secondary transmission from an undiagnosed imported case. This unexpected incident in a new setting occurred during a high transmission period in Togo, which was estimated to have started in December during the previous year, though silently transmitted in the population without early warning. Furthermore, this unusual epidemic was caused by a strain of the virus, later found to be a new lineage by genomic sequencing.

The end of epidemics can be marked by notable and reported changes in the response including official public health declarations, especially for highly pathogenic threats such as Ebola. During outbreak monitoring, the longevity of periods without new cases can be interpreted as a positive sign of improvements in disease control. For the unprecedented epidemic of Mpox in Nigeria during 2017, the first mass incidence in the country, the year of 2018 followed with further declines in reporting, although exported cases were reported in UK and Israel for the first time. By 2019, while monitoring of this epidemic may have been closed due to the decline in reports and declarations, another exported case from Nigeria was unusually detected in Singapore, after 586 days since the Nigeria outbreak was initially reported. This sentinel event was also the first case in a new region of the world and a recurrent warning sign of a potential, under-reported emerging threat; however difficult to diagnose, and track due to the lower mortality.

**Fig. 1:**
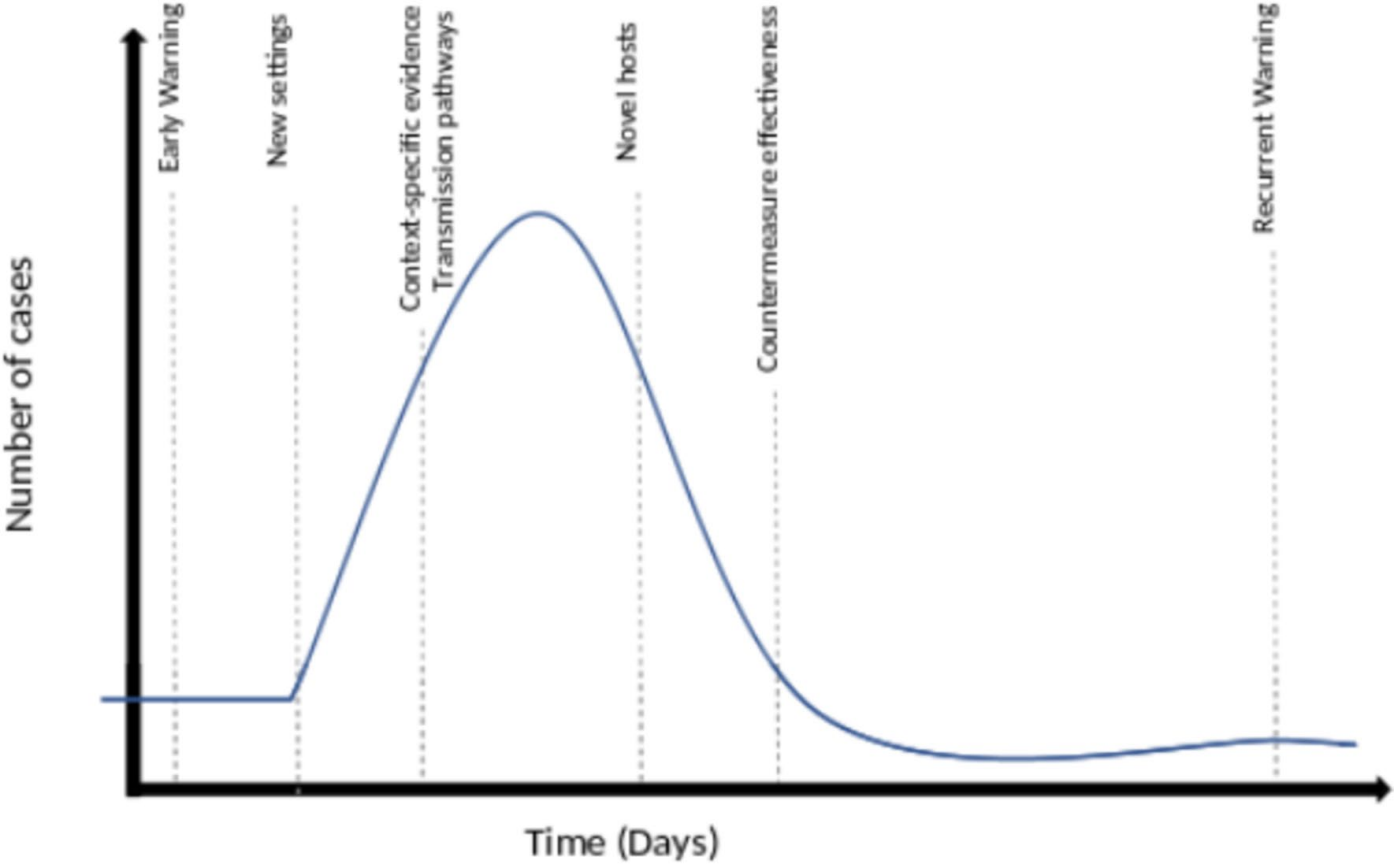
Phases of outbreak evolution and granulation of distinct trigger points gathered from EBS during exceptional, multi-country outbreaks in 2010–19 from spillover to epidemics to subsequent wave

While these alerts gathered from ProMED mail posts were rapid, they are incomplete about the entire unusual and unique disease activity occurring over the decade. With improved reporting in certain countries with high spillover and poor digital reporting sources, the breath of reports will likely to be more comprehensive. A diagram of the observed types of exceptional event triggers observed during phases of an epidemic are summarized in Fig. 1.

Other important triggers, for example, changes in case fatality rates due to increased virulence, are not clearly marked by postings with obvious gaps in timeliness and are not considered in this review. Among the other major disease events during the decade, such as wild or vaccine-derived Poliomyelitis virus (*n* = 362) and Chikungunya virus (*n* = 382) with high numbers of reports observed, and in many affected countries, lacked the markings of significant triggers on information elements from Epidemic Intelligence, after the initial warnings. Wild Polio cases resurfaced again in Nigeria after many years of no reported disease transmission. While this event was unexpected and alarming for eradication efforts, no explanation was given later, which cited extraordinary determining factors. For Chikungunya, after the early signals about first detections in new regions, particularly the Pacific and Americas, few clear and distinct follow-up events were recorded for this review. The virus’s rapid spread and acute illness were well recognized features in early warning. Approval for an effective vaccine for Chikungunya was still in progress following the study period from 2010–19. In these situations, the timeliness of these lapses of information were not substantial in length to distinguish for estimation of improvements beyond early warning from EBS. Similar, challenging global threats like cholera (*n* = 636), with notable events such as re-introduction in Haiti and successful mass vaccination campaigns in Africa, did not have follow-up reports about new and unusual epidemiologic findings influencing the dynamics. Also, an absence of valuable response insights were noted regarding “acute flaccid myelitis” / Enterovirus-D68 reports from 2014–19 in the Western Hemisphere, Rift Valley Fever in South Sudan and Uganda, (re)introduction of Dengue in multiple countries, nor drug-resistant Typhoid and the intercontinental spread of other antimicrobial resistance threats.

## Discussion

Unlike the early warning systems of natural disasters with an abrupt and violent acute phase, such as tsunamis and hurricanes, infectious disease outbreaks may develop further after critical early warning functions and have additional information to enhance the making of predictions to benefit the preparations in future phases [51]. For most disease outbreaks, epidemiological situation updates are a common feature during the response, with at a minimum, the basic information about the number of cases, demographic information, and testing. In simple terms, the numbers generate the latest knowledge about the disease transmission and have more recently been applied for forecasting estimation with the useful intention of predictions. While these indicators are undoubtedly essential and important, there are other dimensions to consider of which the inclusion of details on new changes creating unexpected trajectories offer more nuanced interpretability for complex situations. Since the aim is for an early end to all new outbreaks, once the pathogen becomes established in the setting, overwhelming containment efforts, it can propagate to transmit in multiple countries, the challenges mount outside of preconceptual assessments and renewed interpretation of information should remain constant. Technical information about important triggers are infrequent and sometimes more difficult to update due to the uncategorizable format, unlike a routine epi report with case numbers and testing results. These trigger events can be under-reported and/or investigated early in outbreaks and better followed through EBS approaches. Earlier monitoring and reporting of trigger events will be useful for control of expanding outbreaks.

Furthermore, due to the lack of scientific information during the initial phases of an outbreak of an emerging disease, there is little empirical evidence to base assessments for public health responses. The findings about viral persistence and new species hosts only happen from wider spread, but these unique outbreaks do not have to reach this size with more effective control. Given prolonged lengths of time for the trigger points, shown in this review, the role of EBS is crucial and should be structured above the reactive component of a specific health emergency response, rather within normal operations in anticipation of further emergence related to, though not bound, to early warnings. It is necessary to overcome past preconceptions about how outbreaks will unfold. These insights were rare in perspective among all outbreak events on ProMed mail, but consistent in occurrence across the years showing that careful monitoring is required in dedicated EBS operations. There would be great value in institutionalizing and integrating proactive EBS as an essential function of integrated health systems, building and utilizing reporting systems, such as ProMED or aggregation platforms such as WHO Epidemic Intelligence from Open Source (EIOS) system, and digital optimization tools to strengthen the timely detection of important triggers [52, 53]. In addition, leveraging community-level or health facility-based EBS reporting structures would benefit country-level responses though primarily to the national level and not publicly-accessible. The reports illustrate that emerging microbes remain an unpredictable threat unless completely eradicated, as nearly approaching for Guinea Worm, and will continue to evolve and adapt to new environments.

Corresponding with this changing landscape in outbreak response approaches, the growing threat of new emerging diseases due to environmental and anthropomorphic factors has been much covered in previous reviews [54–56]. This trend underscores the need for further information on outbreak dynamics through increasingly granular data and updates, which provide new insights and allow knowledge production in the multi-sectorial response field. As well, the ability to obtain rapid information granules through internet technology has shifted from the historical priorities of using one common message of warning, followed by less frequent updates on the quantitative changes and the latest scientific insights. In this review of the global outbreak response experience, arguably, there are other granules with wide implication for the outbreaks assessed, though they are not available to include here due to under-reporting, capabilities, and perceived lack of public interest in sharing the findings. As the COVID-19 pandemic highlighted the increasing need for details on population-specific outbreak data, the continuously-evolving scientific knowledge has led to remarkable gains in understanding disease epidemiology and the rapid development of new medical counter-measures such as emergency-approved RNA vaccines [2]. Even the best information and response approaches did not predict the long term consequences of infection characterized as Long COVID-19 [57]. And we have seen in past global health events, there were contemporaneous epidemics, such as Encephalitis lethargica, arising during the 1918–19 Great Influenza Pandemic, remaining poorly understood from the lack of description through rapid communications and adapted reporting style [58, 59].

While these findings pertain to major outbreaks of global interest, the processing of information with greater granularity may be applied for smaller outbreaks at local levels for conceivable gains in nuanced analysis and supporting research interests. For example, triggers on public health control measures, misinformation (such as reported adverse events from vaccination), public trust at large, and high-visibility incidents may be refined as guiding indicators about the performance in community response. For a more thorough command of the emergency and improved public health response, the Public Health EOC of a country may learn to adapt the different settings for a more specialized approach in the sub-populations at higher risk. The review highlights important policy implications for pandemic preparedness and response. It emphasizes the need to shift from reactive to proactive approaches by incorporating EBS into routine process of integrated health systems that would capture new changes and unexpected outbreak trajectories. Trigger detection, confirmation, assessment, reporting and notification mechanisms should be strengthen as required by IHR and are essential for sustainable understanding of outbreak dynamics and informing multi-sectorial and global response efforts. Improving reporting and sharing of outbreak information, including non-scientific triggers, can guide specialized approaches in high-risk sub-populations. Policy efforts should anticipate the evolving nature of outbreaks and embrace an anticipatory EBS approach to adapt to changing circumstances. Continued learning and adaptation are necessary due to the rapid viral evolution and the potential for shifting dynamics in outbreaks. Nevertheless, early warning remains a vital surveillance function, yet must be complemented with ongoing EBS for granules about what is driving the dynamics of internationally-important outbreaks.

For this observational study, the reliance on information from formal reports has several limitations, including the delay in dissemination due to review processes and scheduled release of updates in less timely manner. Additionally, it is possible that other important findings were not made available in formal reports and posted for the public, leading to potential bias in the observed key uses of EBS for outbreaks during the study period. Presumably, other triggers were known within health institutions during their response though were not captured here, the study suggests that outbreak response could be improved through more comprehensive, transparent, and timely scientific reporting, as well as the rapid dissemination of information.

## Conclusions

In conclusion, the outbreak experiences during the past decade benefitted from unanticipated technical information worthwhile after the early warning period. While the triggers captured in this review were likely known much earlier informally, the formal recognition of it, either in official or peer-reviewed postings, is necessary in order to get additional response funding and apply widely for the response. Arguably earlier warnings on these events may not have prevented additional cases due to the unforeseen changing nature of the disease outbreak dynamics. Future responses should aim to ascertain the essential information relevant to these types of developments. An anticipatory EBS approach for these types of triggers seems prescient, as highlighted by these exceptional outbreaks. We continue to learn more as infections increase in a population. As well, with continual and rapid microbial evolution during outbreaks, what is true one day, may not necessarily be true the next days. The role of automated default warnings through existing EBS channels and Artificial Intelligence (AI) in anomaly detection has to be further explored and capitalized upon.

## Data Availability

The datasets generated and/or analysed during the current study are available on the public websites of the ProMed search database and Disease Outbreak News websites (https://promedmail.org/promed-posts/; https://www.who.int/emergencies/disease-outbreak-news). All data generated or analysed during this study are included in this published article in the written text and Table 1.
